# A gastrointestinal stromal tumor at the esophagogastric junction successfully treated by laparoscopic wedge resection with seromuscular layer dissection: a case report

**DOI:** 10.1186/s40792-015-0090-9

**Published:** 2015-09-25

**Authors:** Hidefumi Shiroshita, Norio Shiraishi, Yuki Shitomi, Tsuyoshi Etoh, Seigo Kitano, Masafumi Inomata

**Affiliations:** Department of Gastroenterological and Pediatric Surgery, Faculty of Medicine, Oita University, 1-1 Idaigaoka, Hasama Machi, Yufu, Oita 879-5593 Japan; Center for Community Medicine, Faculty of Medicine, Oita University, 1-1 Idaigaoka, Hasama Machi, Yufu, Oita 879-5593 Japan; Oita University, 1-1 Idaigaoka, Hasama Machi, Yufu, Oita 879-5593 Japan

**Keywords:** Anti-reflux, Gastric GIST, Laparoscopic wedge resection

## Abstract

Herein, we report a case of a gastrointestinal stromal tumor (GIST) at the esophagogastric junction (EGJ) that was successfully treated by a laparoscopic wedge resection (LWR) after dissection of the seromuscular layer around the tumor to prevent postoperative deformities and stenosis of the EGJ. Subsequently, the abdominal esophagus was wrapped by the gastric fornix according to Dor’s method in order to prevent reflux esophagitis after surgery.

A 71-year-old female patient was admitted with a diagnosis of a GIST (23 × 20 × 20 mm) at the EGJ. We performed the abovementioned operation.

Gastroduodenal endoscopic examination revealed no deformity or stenosis of the EGJ at 6 months after the operation. The patient has not experienced any reflux symptoms. Tumor recurrence was not noted 26 months after the operation.

This procedure is useful in preventing the deformity and stenosis of the EGJ as well as postoperative reflux esophagitis.

## Background

Gastrointestinal stromal tumor (GIST) is the most common mesenchymal neoplasm of the gastrointestinal tract. About 60 % of all GISTs occur in the stomach, 30 % in the small intestine, and 10 % in the esophagus, colon, and rectum [1]. The malignant potential of GISTs is classified according to tumor size and mitotic activity [2–4]. Surgical resection is the standard treatment for GIST. The frequency of lymph node metastasis in GIST is low [5]; therefore, local resection without lymph node dissection is commonly used as the standard treatment for GIST.

Recently, laparoscopic wedge resection (LWR) is being performed with greater frequency for the treatment of GIST of the stomach because it is less invasive than other procedures. Furthermore, single incisional laparoscopic local gastrectomy for gastric GIST has been reported [6]. However, as much of the normal gastric wall around the tumor may be removed in LWR for GIST, severe deformity and stenosis of the stomach and disturbance of gastric function often occur after LWR, especially for GIST at the esophagogastric junction (EGJ). Recently, combined laparoendoscopic approaches, such as laparoscopy-endoscopy cooperative surgery (LECS) and endoscopic full thickness resection (EFTR), have been developed in order to prevent postoperative deformities and stenosis of the EGJ [7–9].

We report a case of a gastric GIST at the EGJ that was successfully treated by LWR with Dor’s fundoplication after dissection of the seromuscular layer around the tumor.

## Case presentation

A 71-year-old female patient was admitted to our hospital with a gastric mass incidentally identified by gastroduodenal endoscopic examination during screening. No physical abnormalities were observed. Laboratory tests including hematologic and biochemical analyses revealed no abnormalities.

**Fig. 1:**
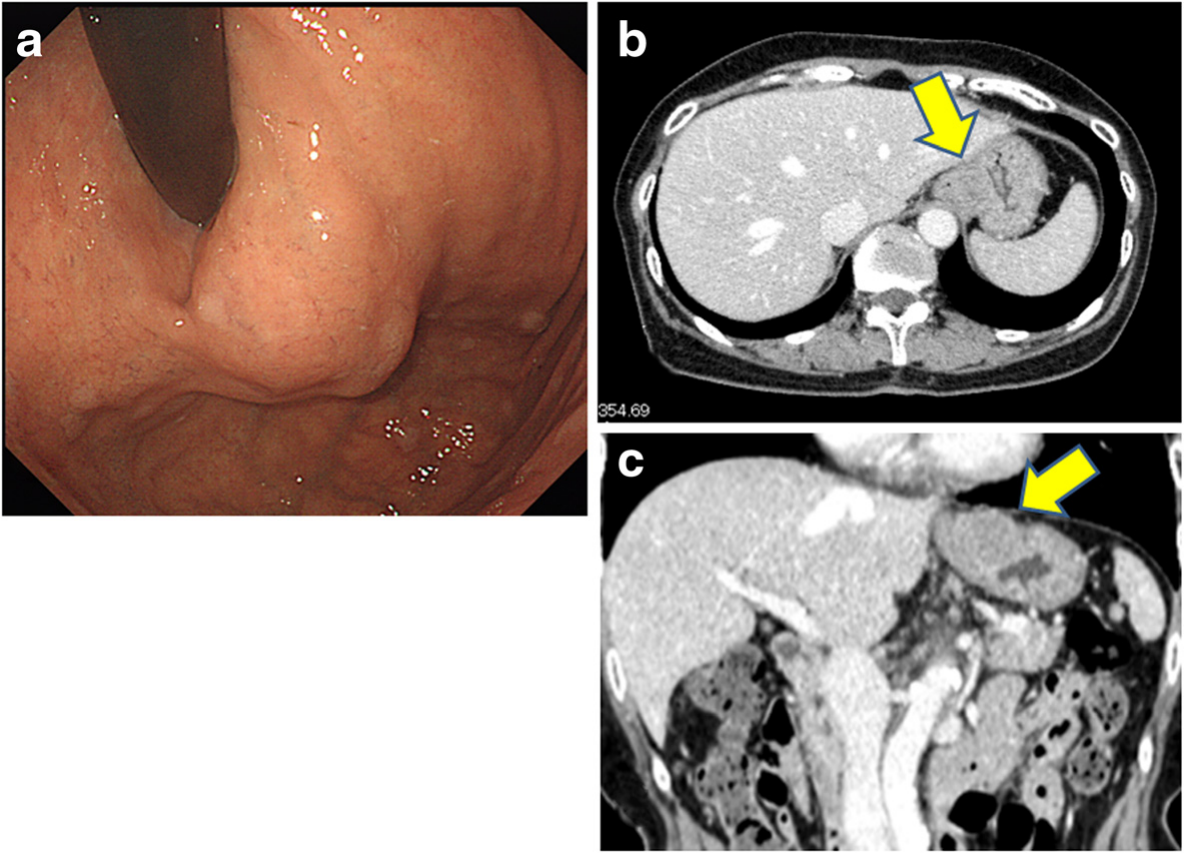
**a** Gastroduodenal endoscopy revealing the presence of a submucosal tumor at the greater curvature of the esophagogastric junction. **b, c** The enhanced computed tomography (CT) scan shows a smooth-outlined hypervascular solid mass (23 mm × 20 mm) in the gastric wall at the esophagogastric junction (*arrow*)

A gastroduodenal endoscopic examination revealed the presence of a submucosal tumor at the greater curvature of the EGJ (Fig. 1a). An enhanced computed tomography scan revealed a solid mass (23 × 20 mm) with a smooth margin and hypervascularity in the gastric wall at the EGJ (Fig. 1b, c). Examination by endoscopic ultrasonography showed the presence of a hypoechoic submucosal nodule at the greater curvature of the EGJ. A fine-needle aspiration was performed, and the pathologic diagnosis of the submucosal tumor was a possible gastric GIST.

**Fig. 2:**
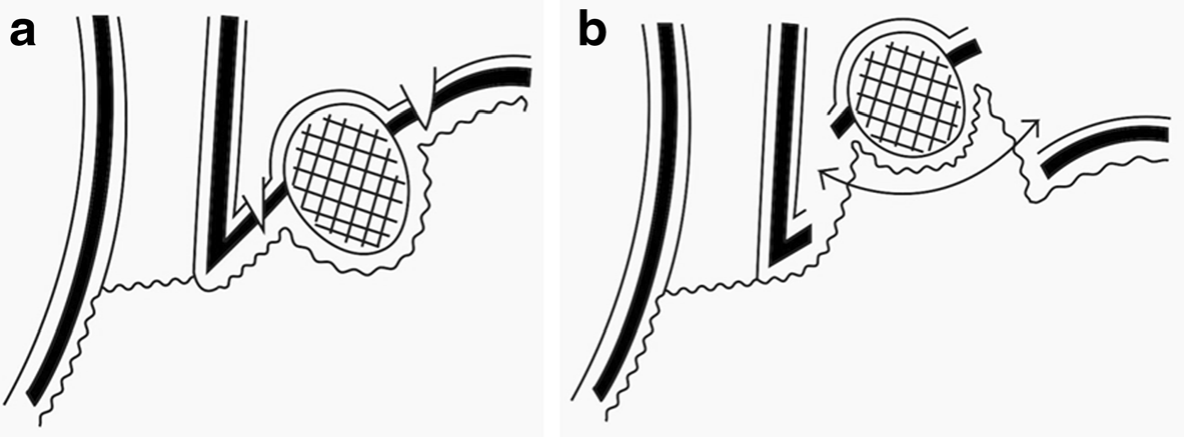
Schema of LWR. **a** Seromuscular layer was dissected. **b** Mucosal layer was cut by linear stapler

We performed LWR after laparoscopic dissection of the seromuscular layer around the tumor to prevent postoperative deformities and stenosis of the EGJ. Our procedure minimized the area of the gastric wall to be removed and did not expose the peritoneal cavity to any intragastric content (Fig. 2).

**Fig. 3:**
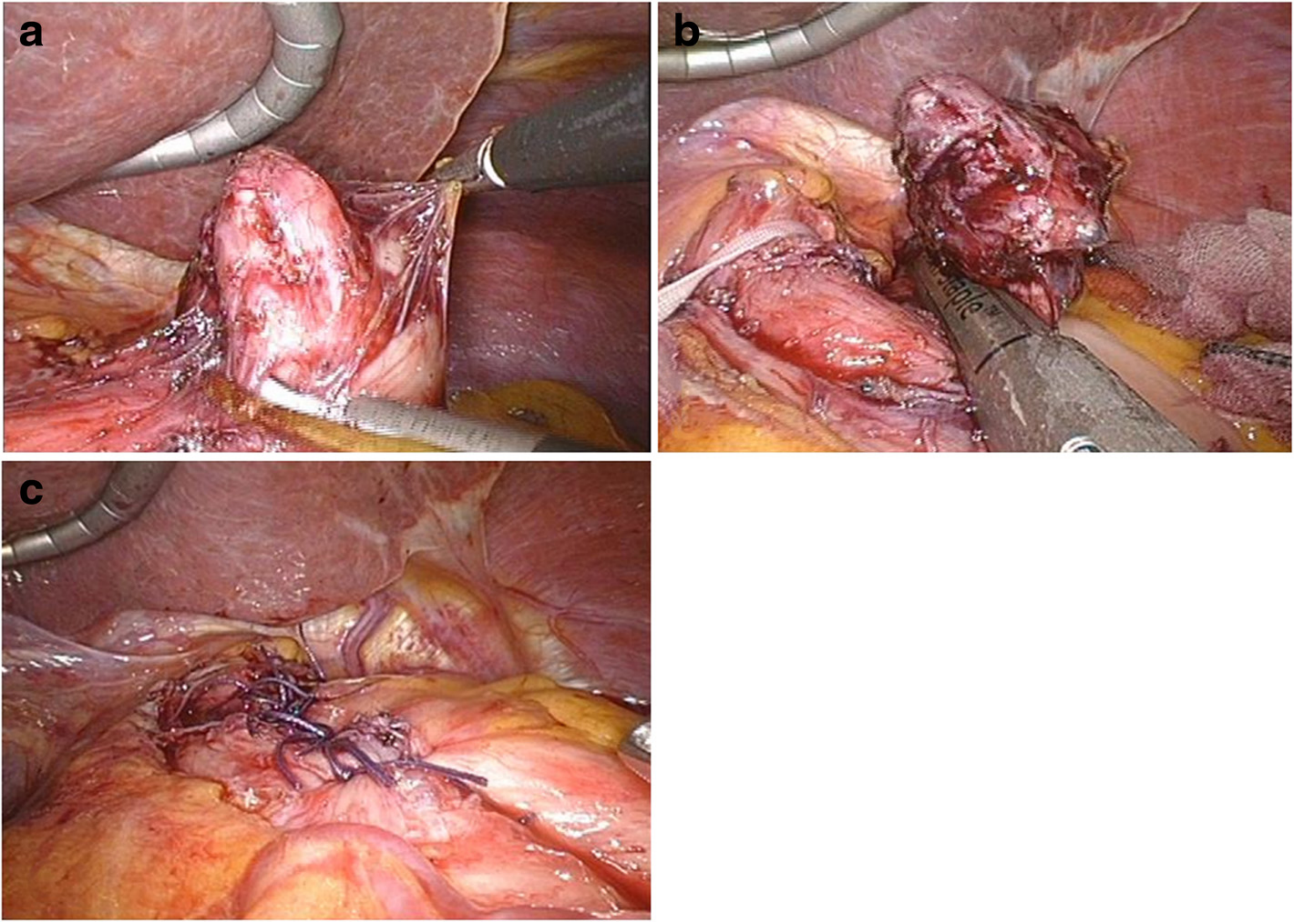
**a** The adventitia of the abdominal esophagus and seromusucular layers are separated in order to view the tumor base. **b** Once the tumor is mobilized, a laparoscopic wedge resection of the gastric lesion is performed with an endo GIA linear stapler (Endo GIA™ tri-stapler, with two purple cartridges). **c** The fornix is wrapped around the abdominal esophagus following Dor’s procedure in order to reinforce the resected portion and prevent reflux

After a 12-mm trocar was placed at the umbilicus, the laparoscope was inserted into the abdominal cavity. Under laparoscopic view, two additional ports (10 mm) were placed at both outer edges of the right and left abdominal rectus muscle in the upper abdomen and one additional 5-mm trocar was inserted in the subcostal region. The tumor was easily identified at the EGJ as a protruding mass covered with adipose tissue. Mobilization of the EGJ was accomplished laparoscopically. We exposed the anterior wall of the abdominal esophagus and revealed a tumor attached to its left wall. We isolated the adventitia of the abdominal esophagus and seromuscular layer of the stomach and identified the tumor base (Fig. 3a). After dissection of the seromuscular layer around the tumor, the tumor was able to be lifted using atraumatic grasping forceps.

The LWR for the gastric GIST was performed with an Endo GIA linear stapler (Endo GIA™ tri-staple, with two purple cartridges) (Fig. 3b). Then, the exposed front surface of the abdominal esophagus was wrapped by the gastric wall of the gastric fornix according to Dor’s method to reinforce the dissected part of the EGJ and prevent reflux esophagitis (Fig. 3c).

The resected specimen was extracted through the umbilical trocar site using an Endocatch bag. The operation time was 171 min. Gross findings of the tumor resected by LWR showed that the tumor capsule was neither injured nor ruptured during surgery.

By pathologic examination of the resected specimen, the tumor was diagnosed as a gastric GIST with a negative margin. The dimension of the tumor was 26 × 24 × 24 mm. The tumor was found to be composed of spindle-shaped tumor cells. Immunohistochemical examination showed that the tumor cells were strongly positive for CD34, c-kit, and DOG1; a weakly positive for alpha-smooth muscle actin; and negative for S100. The mitotic count was just 1/50 HPF; therefore, according to the Fletcher Risk Table, the GIST was classified as a low-risk malignancy.

**Fig. 4:**
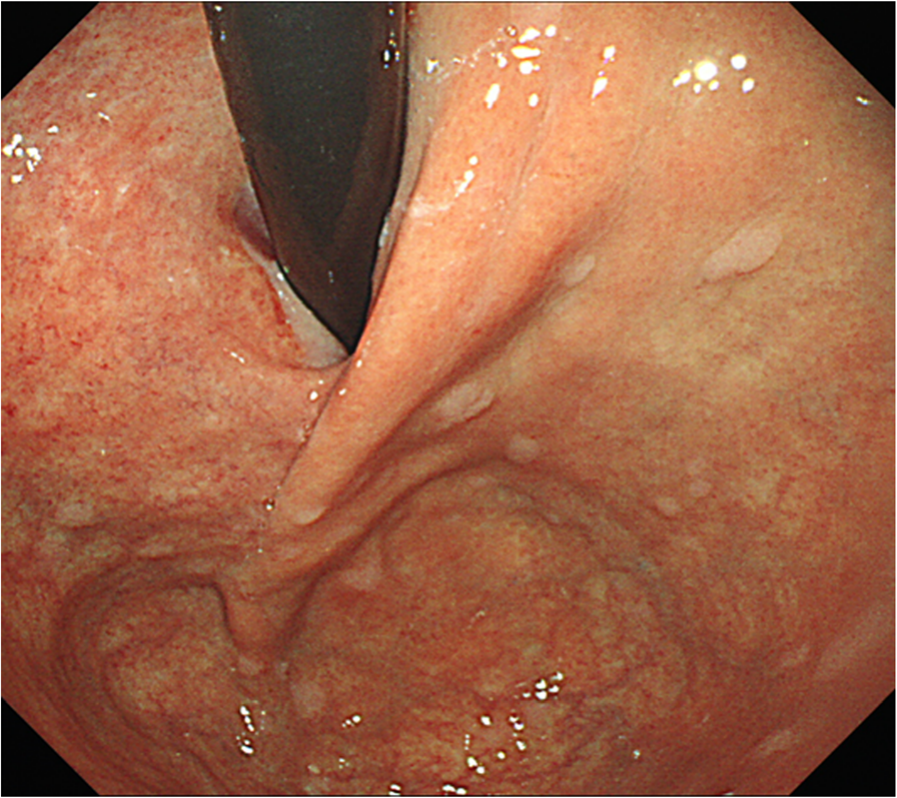
A gastroduodenal endoscopy 6 months postoperation shows the absence of stenosis in the esophagogastric junction

There were no postoperative complications, and the patient was discharged per her request 10 days after the operation. Gastroduodenal endoscopic examination revealed no deformity or stenosis of the EGJ at 6 months after the operation (Fig. 4). The patient has not experienced any reflux symptoms. Furthermore, there was no evidence of GIST recurrence by either abdominal ultrasonography or computed tomography during the 26-month follow-up period.

### Discussion

GISTs originate from the gastrointestinal wall as submucosal tumors. In GIST, the frequency of lymph node metastasis is low. LWR is commonly used for the treatment of gastric GISTs because it is less invasive than open surgery [3]. Despite this, the size of specimen resected by LWR is larger than that resected in open surgery because of technical limitations in wedge resection of the gastric wall by use of a linear stapler. Hence, postoperative complications of severe stenosis and deformity of the stomach could occur after LWR. Furthermore, when the GIST is located at the EGJ, postoperative complications that disturb food flow and cause reflux esophagitis occur after LWR. Thus, LWR as a surgical strategy for gastric submucosal tumors close to the EGJ is still controversial.

Three issues must be resolved for LWR to become the standard procedure for GIST close to the EGJ: (1) how to prevent the disturbance of food flow after LWR, (2) how to protect the abdominal cavity from exposure to intragastric contents during the procedure, and (3) how to prevent reflux esophagitis after the procedure.

With LWR for GISTs close to the EGJ, the resected specimen tends to be large because of limitations in the manipulation range of the stapling device. The primary goal of surgical treatment for GISTs is to achieve resection of the tumor with a negative margin [10]. Recently, combined laparoendoscopic approaches, such as LECS and EFTR, have been developed to avoid stenosis and deformity of the gastric cardia after surgery [7–9]. In addition, percutaneous endoscopic intragastric surgery (PEIGS) has been reported for gastric submucosal tumors [11]. These surgical procedures may be useful for intragastric growth-type GIST located at the posterior wall. In our case, the GIST showed extragastric growth and was located at the EGJ. Therefore, we performed LWR after dissecting the seromuscular layer around the tumor to prevent severe stenosis and cause as little deformity to the EGJ as possible.

Both combined laparoendoscopic approaches and PEIGS involve the risk of exposing intragastric contents to the abdominal cavity. These intragastric contents may contain malignant cells and bacteria. Waterman et al. reported a case of peritoneal dissemination of GIST during surgery [12]. Therefore, for the treatment of GIST close to the EGJ, we used the LWR procedure in which both resection of the gastric wall and suturing were performed at the same time to prevent the dissemination of malignant cells.

The additional surgical procedure to avoid reflux esophagitis after LWR becomes necessary when the GIST is located adjacent to the EGJ. After resecting the tumor at the EGJ, the cardiac sphincter system and the angle of His may be destroyed. In our case, the muscle layer of the abdominal esophagus was exposed to mobilize the GIST. We wrapped the front surface of abdominal esophagus with the gastric wall of the gastric fornix, and Dor’s method, rather than Nissen’s or Toupet’s method, was used to reinforce the exposed front surface of the abdominal esophagus and to prevent reflux esophagitis. The patient did not experience any reflux symptoms after surgery.

## Conclusions

Herein, we report the case of a gastric GIST at the EGJ that was successfully treated by LWR after laparoscopic dissection of the seromuscular layer. This procedure is useful for the treatment of GISTs at the EGJ.

## Consent

Written informed consent was obtained from the patient for the publication of this case report and any accompanying images. A copy of the written consent is available for review by the Editor-in-Chief of this journal.

## References

[1] Miettinen M, Sarlomo-Rikala M, Lasota J (1999). Gastrointestinal stromal tumor: recent advances in understanding of their biology. Hum Pathol.

[2] Dematteo RP, Gold JS, Saran L, Gönen M, Liau KH, Maki RG (2008). Tumor mitotic rate, size, and location independently predict recurrence after resection of primary gastrointestinal stromal tumor (GIST). Cancer.

[3] Novitsky YW, Kercher KW, Sing RF, Heniford BT (2006). Long-term outcomes of laparoscopic resection of gastric gastrointestinal stromal tumors. Ann Surg.

[4] Gold JS, Gönen M, Gutiérrez A, Broto JM, García-del-Muro X, Smyrk TC (2009). Development and validation of a prognostic nomogram for recurrence-free survival after complete surgical resection of localised primary gastrointestinal stromal tumour: a retrospective analysis. Lancet Oncol.

[5] Matthews BD, Walsh RM, Kercher K, Sing RF, Pratt BL, Answini GA (2001). Laparoscopic vs. open resection of gastric stromal tumors. Surg Endosc.

[6] Takata A, Nakajima K, Kurokawa Y, Takahashi T, Yamasaki M, Miyata H (2014). Single-incision laparoscopic partial gastrectomy for gastric submucosal tumors without compromising transumbilical stapling. Asian J Endosc Surg.

[7] Hiki N, Yamamoto Y, Fukunaga T, Yamaguchi T, Nunobe S, Tokunaga M (2008). Laparoscopic and endoscopic cooperative surgery for gastrointestinal stromal tumor dissection. Surg Endosc.

[8] Ikeda K, Mosse CA, Park PO, Fritscher-Ravens A, Bergström M, Mills T (2006). Endoscopic full-thickness resection: circumferential cutting method. Gastrointest Endosc.

[9] Obuchi T, Sasaki A, Baba S, Nitta H, Otsuka K, Wakabayashi G (2015). Single-port laparoscopic and endoscopic cooperative surgery for a gastric gastrointestinal stromal tumor: report of a case. Surg Today.

[10] Das A, Wilson R, Biankin AV, Merrett ND (2009). Surgical therapy for gastrointestinal stromal tumors of the upper gastrointestinal tract. J Gastrointest Surg.

[11] Kanehira E, Kamei A, Umezawa A, Kurita A, Tanida T, Nakagi M. Long-term outcomes of percutaneous endoscopic intragastric surgery in the treatment of gastrointestinal stromal tumors at the esophagogastric junction. Surg Endosc 2015; Epub ahead of print. 10.1007/s00464-015-4439-8 26201418

[12] Waterman AL, Grobmyer SR, Cance WG, Hochwald SN (2008). Is endoscopic resection of gastric gastrointestinal stromal tumors safe?. Am Surg.

